# Revealing the effect of sea buckthorn oil, fish oil and structured lipid on intestinal microbiota, colonic short chain fatty acid composition and serum lipid profiles in vivo

**DOI:** 10.1007/s13659-024-00461-z

**Published:** 2024-07-03

**Authors:** Ankang Song, Yanbo Li, Wei Wang, Yueqi Hu, Junjie Xu, Zhixin Xu, Li Zhou, Jikai Liu

**Affiliations:** 1 College of Food Science and Pharmacy, Xinjiang Agricultural University, Urumqi, 830000 People’s Republic of China; 2 National Demonstration Center for Experimental Ethnopharmacology Education, School of Pharmaceutical Sciences, South-Central MinZu University, Wuhan, 430074 People’s Republic of China

**Keywords:** Dietary lipids, Serum lipid profile, Short chain fatty acid, Intestinal microbiota

## Abstract

In this study, the effects of sea buckthorn oil (SBO), fish oil (FO) and an enzymatically synthesized structured lipid (SL) on serum, short-chain fatty acids (SCFAs) and intestinal microbiota in Sprague–Dawley (*SD*) rats were investigated. The results demonstrated that FO, SBO, and SL effectively reduced the levels of high-density lipoprotein cholesterol and low-density lipoprotein cholesterol in the serum of *SD* rats. SBO increased serum triglyceride levels, while FO elevated total cholesterol levels. Furthermore, all three dietary lipids decreased short-chain fatty acid production and enhanced intestinal microbiota diversity. FO increased the abundance of intestinal microbiota including *Romboutsia*, *Lactobacillus*, *Escherichia-Shigella*, and *Lachnospiraceae_NK4A136_group*. Conversely, all three dietary lipids reduced the abundance of *Klebsiella* and *Blautia*. These findings provide a foundation for understanding the functionality of SBO and FO as well as their potential application in synthesizing novel SLs to regulate intestinal microbiota.

**Graphical Abstract:**

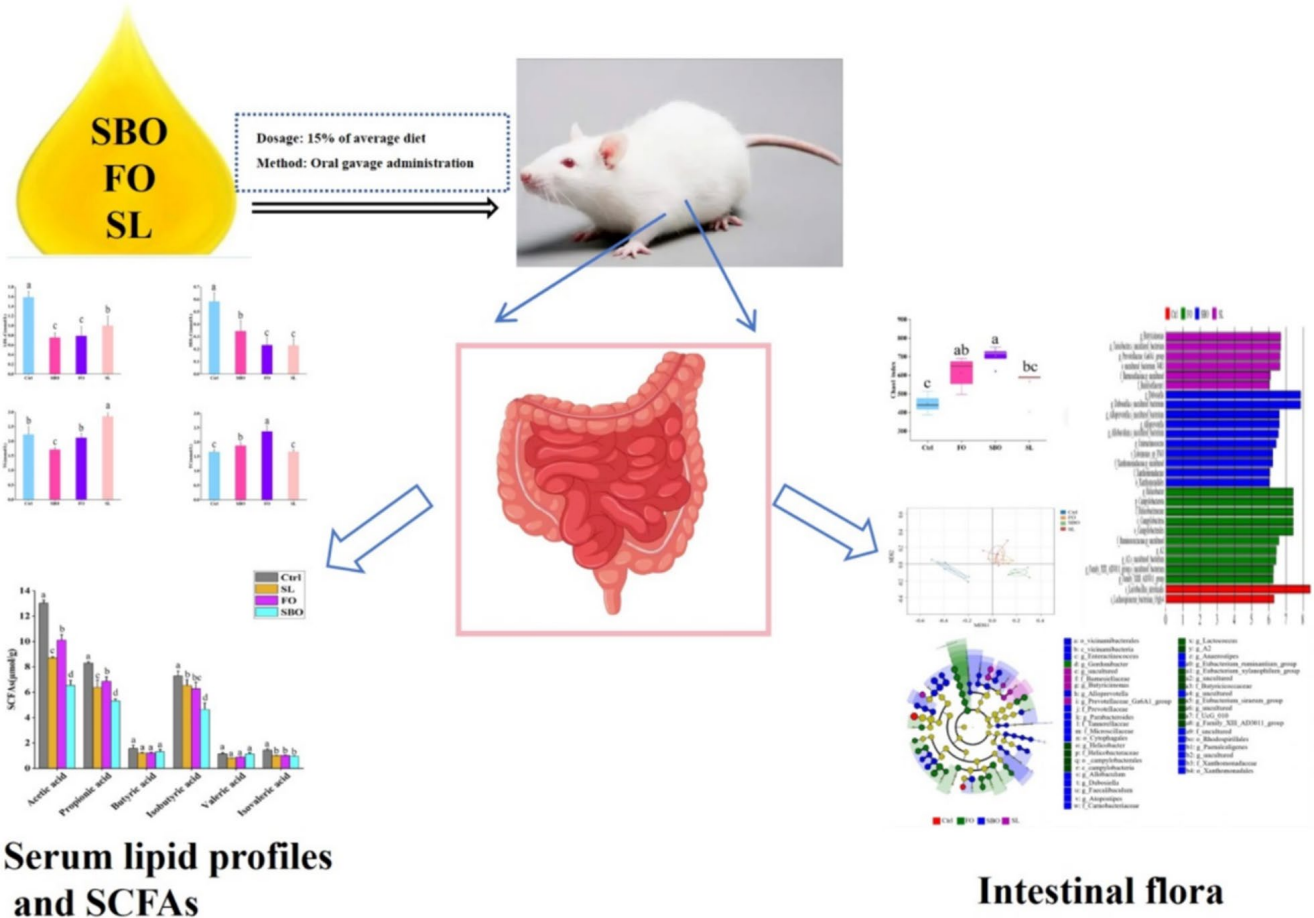

**Supplementary Information:**

The online version contains supplementary material available at 10.1007/s13659-024-00461-z.

## Introduction

Functional oil plays a vital role in the human diet due to its abundant bioactive substances [1]. Dietary fat is an important part of the human diet, and its nutritional value is largely determined by its fatty acid composition, which is closely related to human health [2]. Extensive research has been conducted on functional oil in specialized dietary products and prepared foods. Different types of oils have distinct fatty acid compositions [3]. SBO is derived from the flesh of sea buckthorn and contains lipophilic components such as unsaturated fatty acids, plant sterols, and vitamins A and E, which possess antioxidant, anti-inflammatory, and immunomodulatory effects [4–6]. Previous studies have demonstrated that SBO can modulate intestinal microbiota diversity and composition while enhancing intestinal mucosal and systemic immune function by promoting short-chain fatty acid concentrations [7]. FO is rich in n-3 long chain unsaturated fatty acids, including eicosapentaenoic acid (EPA) and docosahexaenoic acid (DHA) [8, 9]. Both n-3 and n-6 fatty acids are considered essential for the human diet, since they cannot be synthesized within the body, so need to be supplemented from external sources [10]. Numerous studies have indicated that consuming seafood rich in EPA and DHA can alleviate hyperlipidemia, cancer, inflammation, as well as neurological diseases [11–13].

SLs represent a novel category of functional lipids amenable to chemical, enzymatic, or genetic modifications [14]. SLs possess the physiological attributes necessary for enhancing nutritional properties and effectively addressing human health requirements [15]. Initially employed in formulating fats enriched with medium chain fatty acids to alleviate fat malabsorption issues in patients, SLs have been widely applied in medical, food, chemical, and related domains [16, 17]. The gut microbiome, a complex reservoir of metabolites consisting of hundreds of billions of microbes, resides in the gastrointestinal tract [18, 19]. In recent years, there has been a growing research focus on the intestinal microbiota and its crucial role in maintaining host health or triggering various diseases [2, 20]. Imbalances in the structure of the intestinal microbiota can have an impact on the host's metabolic and circulatory systems, with fatty acids playing a significant role as part of gut metabolites [21]. For example, imbalances in the intestinal microbiota have been associated with inflammatory bowel disease, colorectal cancer, obesity, diabetes, atherosclerosis, and liver disease [1, 21–24]. It is widely recognized that diet plays a major role in influencing the composition and function of the human intestinal microbiota which regulates various immune functions within the body. Previous studies have shown that oils rich in unsaturated fatty acids can regulate dyslipidemia caused by high-fat diets through modulation of intestinal microbiota to prevent related diseases. Research has also demonstrated that feeding mice a high-fat and high-sugar diet leads to changes in proportions of intestinal microbiota along with excessive fat storage and disrupted lipid metabolism. Functional oil has been found to regulate body weight and intestinal microbiota in mice. Sea buckthorn seed oil effectively reduces blood cholesterol levels in hypercholesterolemic hamsters by increasing intestinal cholesterol excretion and promoting growth of SCFA-producing bacteria [25]. Whereas SBO can modulate diversity and composition of intestinal microbiota in CTX-induced immunosuppressed Balb/c mice thereby enhancing both mucosal and systemic immune responses [7].

SBO and FO contain a diverse range of bioactive compounds with potential health benefits. However, their impact on the intestinal microbiota remains understudied. Therefore, the effects of SBO, FO and SL (Obtained by enzymatic reaction of SBO and FO) on intestinal microbiota, colonic SCFAs and serum lipid profiles were investigated by establishing an *SD* rat model.

## Results

### Fatty acid composition of lipid samples

The fatty acid composition of the studied lipids was shown in Table 1. The most abundant fatty acids in SBO were C16:0, C16:1, C18:1 and C18:2, accounting for more than 90% of the total fatty acids. The top three fatty acids in FO were C20:5, C22:6 and C18:2, respectively, and the content of EPA and DHA in the total fatty acids exceeds 65%. The highest content of fatty acids in SL were EPA and DHA, and the content of other fatty acids were also relatively rich, among which the content of EPA/DHA accounts for about 44.8% of the total fatty acids.

**Table 1:** Fatty acid composition

Fatty acid	Composition (%)		
	SBO	FO	SL
C14:0	0.76 ± 0.01	0.47 ± 0.03	0.46 ± 0.04
C15:0	0.12 ± 0.00	nd	0.01 ± 0.00
C16:0	23.71 ± 0.23	0.86 ± 0.10	11.63 ± 0.28
C16:1n7	28.43 ± 0.18	0.93 ± 0.03	12.33 ± 0.31
C18:0	1.90 ± 0.06	0.49 ± 0.00	0.52 ± 0.01
C18:1	20.96 ± 0.53	4.55 ± 0.13	13.62 ± 0.47
C18:2n6	19.09 ± 0.81	13.80 ± 0.35	15.99 ± 0.69
C18:3n3	1.16 ± 0.02	1.56 ± 0.02	0.36 ± 0.02
C20:0	0.28 ± 0.00	3.32 ± 0.08	0.23 ± 0.00
C20:2	1.26 ± 0.05	nd	0.01 ± 0.00
C20:4n6	0.01 ± 0.00	3.23 ± 0.31	0.01 ± 0.00
C20:5n3	nd	43.86 ± 0.8	33.65 ± 0.75
C22:0	0.17 ± 0.01	4.52 ± 0.05	0.01 ± 0.00
C22:1	nd	0.15 ± 0.00	0.01 ± 0.00
C22:2	nd	0.11 ± 0.00	nd
C22:4	nd	0.19 ± 0.00	nd
C22:6n3	nd	21.60 ± 0.64	11.15 ± 0.38
C24:0	0.12 ± 0.01	0.10 ± 0.00	0.01 ± 0.00
SFA	27.06 ± 0.42	9.76 ± 0.01	12.87 ± 0.03
UFA	70.91 ± 0.61	89.98 ± 0.78	87.13 ± 0.96
MUFA	49.39 ± 0.28	5.63 ± 0.01	25.96 ± 0.15
PUFA	21.52 ± 0.12	84.35 ± 0.49	66.17 ± 0.38

*SFA* saturated fatty acid, *UFA* unsaturated fatty acid, *MUFA* monounsaturated fatty acid, *PUFA* polyunsaturated fatty acid, *nd* means not detected

### Serum lipid profiles of *SD* rats after 6-week of different dietary lipid intake

**Fig. 1:**
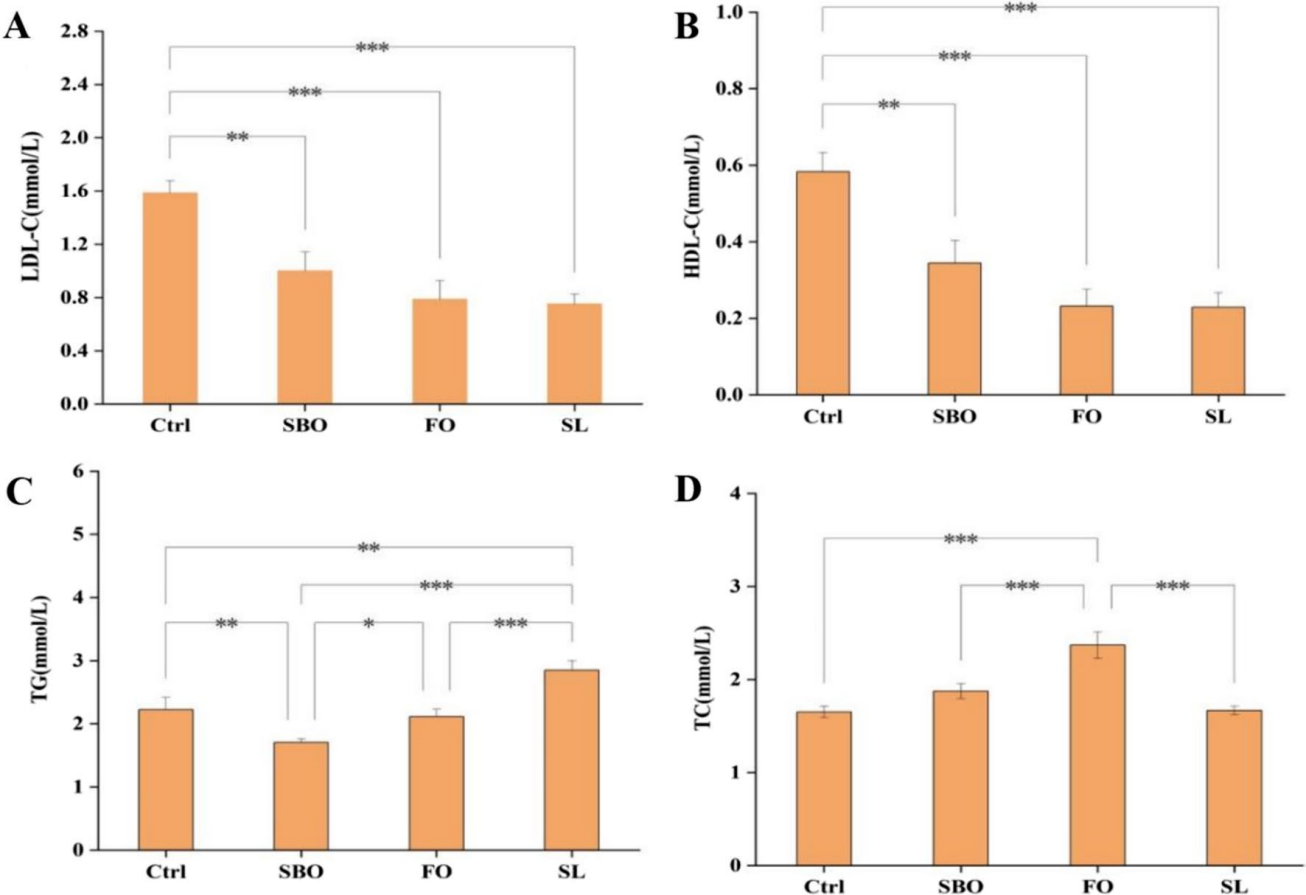
The serum lipid profifiles of the SD rats after 6-week lipid oral gavage administration. **A** Low-Density Lipoprotein Cholesterol; **B** High-Density Lipoprotein Cholesterol; **C** Triglycerides (TG); **D** Total Cholesterol (TC). The significance level was set at 0.05, ***p < 0.001, **p < 0.01, *p < 0.05

After six weeks of treatment with different dietary lipids, there were significant differences in blood lipid levels in *SD* rats (Fig. 1). The serum low-density lipoprotein cholesterol (LDL-C) (Fig. 1A) and high-density lipoprotein cholesterol (HDL-C) (Fig. 1B) concentrations in Ctrl group were significantly higher than those in SL, FO, and SBO groups (*p* < 0.01). The HDL-C concentration in SBO group was higher than that in FO and SL groups. The concentration of serum triglycerides (TG) (Fig. 1C) in SL group was significantly higher than that in Ctrl, SBO and FO groups (*p* < 0.01). The TG concentration in Ctrl and FO groups was significantly different from that in SBO group (*p* < 0.05). The serum total cholesterol (TC) concentration in FO group (Fig. 1D) was significantly higher than that in Ctrl, SL and SBO groups (*p* < 0.001).

### SCFA concentration in colon contents

**Fig. 2:**
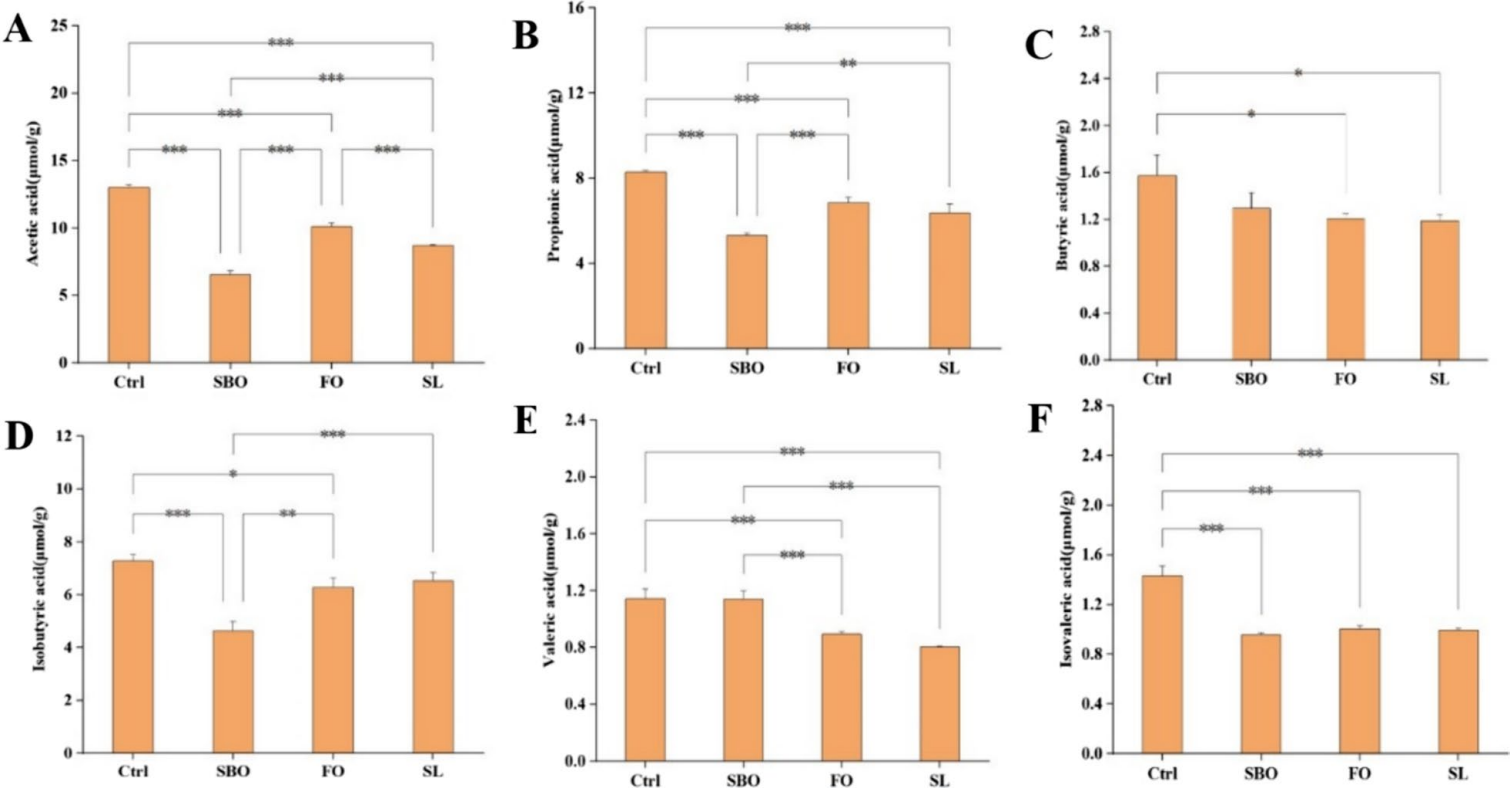
SCFAs concent. **A** Acetic acid; **B** Propionic acid; **C** Butyric acid; **D** Isobutyric acid; **E** Valeric acid; **F** Isovaleric acid. The significance level was set at 0.05, ***p < 0.001, **p < 0.01, *p < 0.05

The concent of total SCFAs in the Ctrl group was higher than that in the different oil feeding groups (Fig. 2). Except for the Ctrl group, acetic acid and propionic acid were the highest in FO group, followed by SL group, and the lowest in SBO feeding group. The concent of butyric acid was not statistically significant between the Ctrl and SBO groups (*p* > 0.05). The isobutyric acid concent of SBO was significantly lower than that of SL and FO groups (*p* < 0.01), and there was no difference between SL and FO groups (*p* > 0.05). There was no significant difference in valeric acid concent among Ctrl and SBO (*p* > 0.05). The content of isovalerate in Ctrl group was significantly different from that in SL, FO and SBO groups (*p* < 0.001), there was no significant difference in isovaleric acid concent among SL, FO and SBO (*p* > 0.05).

### Cecal microbiota diversity

#### Alpha diversity index analysis

**Fig. 3:**
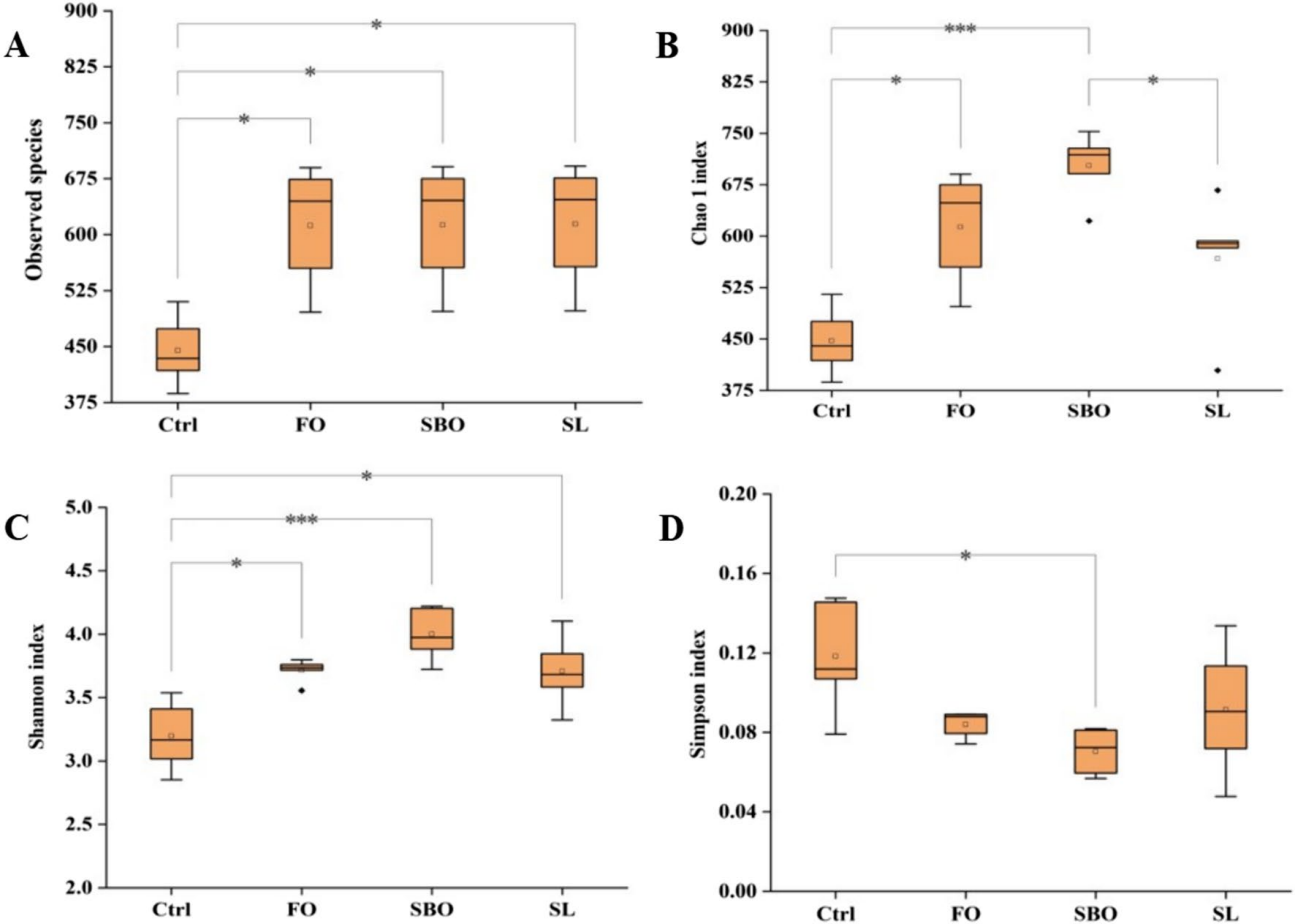
Alpha diversity index for each group. **A** Observed species; **B** Chao 1 index; **C** Shannon index; **D** Simpson index. The significance level was set at 0.05, ***p < 0.001, **p < 0.01, *p < 0.05

The Observed species and Chao1 indices reflect the abundance of species, while the Shannon index and Simpson index reflect species diversity. As depicted in Fig. 3, the intestinal microbiota communities of FO and SBO groups exhibit higher values for both Observed species (Fig. 3A) and Chao1 (Fig. 3B) indices compared to the Ctrl group. Moreover, there were no significant differences in microbial diversity between SBO and FO groups, FO and SL groups. The Observed species and Chao1 indicated that the intestinal microbial communities in SBO and FO groups were significantly differ from those in the Ctrl group (*p* < 0.01). Furthermore, although there were no significant differences observed in the Shannon index (Fig. 3C) or Simpson index (Fig. 3D) among FO, SBO, and SL groups (*p* > 0.05), it is noteworthy that the Shannon index was highest in the SBO group followed by SL and FO groups, respectively, whereas the Simpson index was lowest in the SBO group followed by FO and SL.

#### Beta diversity analysis

##### Principal coordinate analysis (PCoA)

**Fig. 4:**
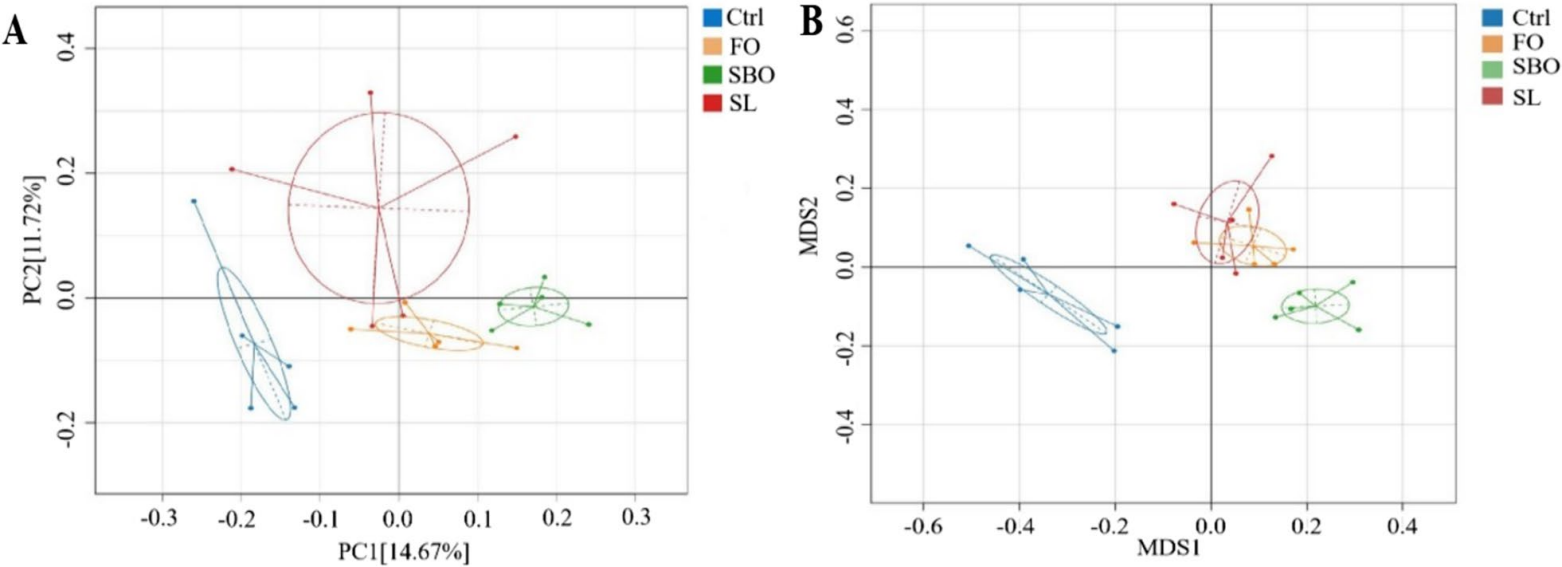
**A** Weighted Unifrac algorithm PCoA analysis; **B** Weighted Unifrac algorithm NMDS analysis

The weighted Unifrac algorithm was used in this study to comprehensively consider both species presence and abundance. Abundance was then weighted to calculate the distance between samples, allowing for observation of population differences between different samples or populations. As shown in Fig. 4A, the first principal component contributed 14.67% to the difference between samples, while the second principal component contributed 11.72%. The results showed that different dietary lipids altered the intestinal microbiota of *SD* rats, which was significantly different from that of the Ctrl group. The intestinal microbiota of the groups treated with different dietary lipids and the Ctrl group were distinctively separated. Moreover, in terms of spatial distribution, the SL processing group exhibited closer similarity to the Ctrl group.

##### Nonmetric multidimensional scaling (NMDS) analysis

The results of weighted Unifrac nonmetric scaling show that each point in the figure represents a sample, while different colors of the points were used to represent groups of samples. The closer the distribution of points, the more similar the samples were represented. There was significant clustering of microbial communities among different groups. The results indicated significant differences between the Ctrl group and the FO, SBO and SL treated bacterial communities (Fig. 4B).

##### Heatmap analysis

A heatmap was generated using a distance algorithm and the R language tool (Additional file 1: Fig. S1). The similar samples were clustered closer together in the tree, which reflectd the similarity and difference in species composition at the genus level for all samples. The color represented the abundance of species, with a gradient from blue to red indicating increasing abundance.

### Microbiota composition of cecum

#### Venn diagram analysis of species

**Fig. 5:**
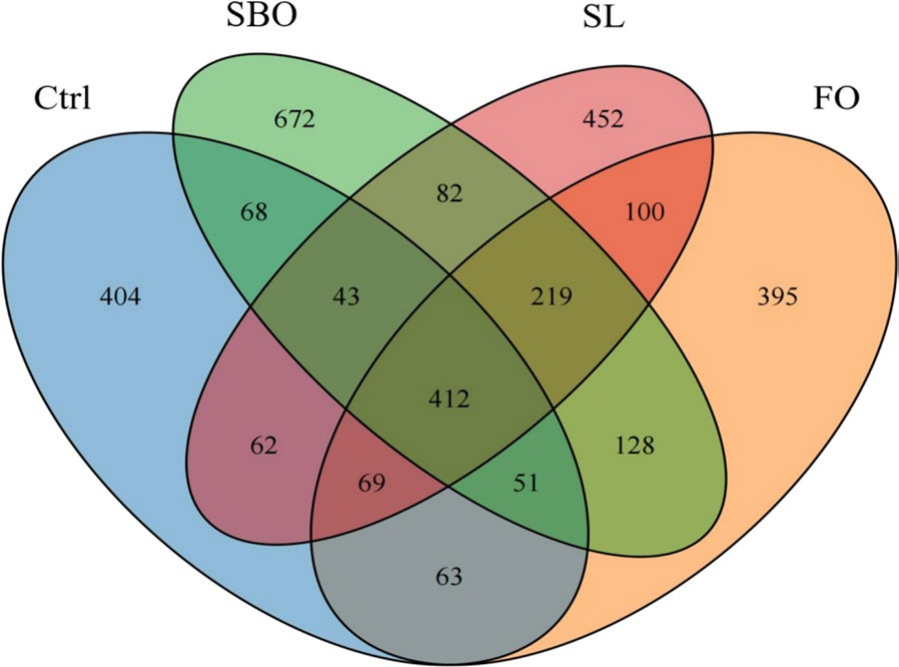
Venn diagrams of OUT distribution in all 4 groups

In order to investigate the regulatory effects of different dietary fats on intestinal microbiota, we employed high-throughput sequencing of bacterial 16S rRNA to determine the composition of the bacterial community in the sample. Therefore, we sequenced the original cecal microbiota of rats fed different dietary lipids for 6 weeks. Based on the analysis of Fig. 5, a total of 412 shared operational taxa (OTUs) were observed across all four groups, indicating a presence of at least 412 species of symbiotic bacteria in these groups. The number of specific OTUs in Ctrl, FO, SBO and SL groups were found to be 404, 395, 672 and 452 respectively, suggesting distinct microbial species associated with each component.

#### Analysis of community variability between groups

##### Phylum level

**Fig. 6:**
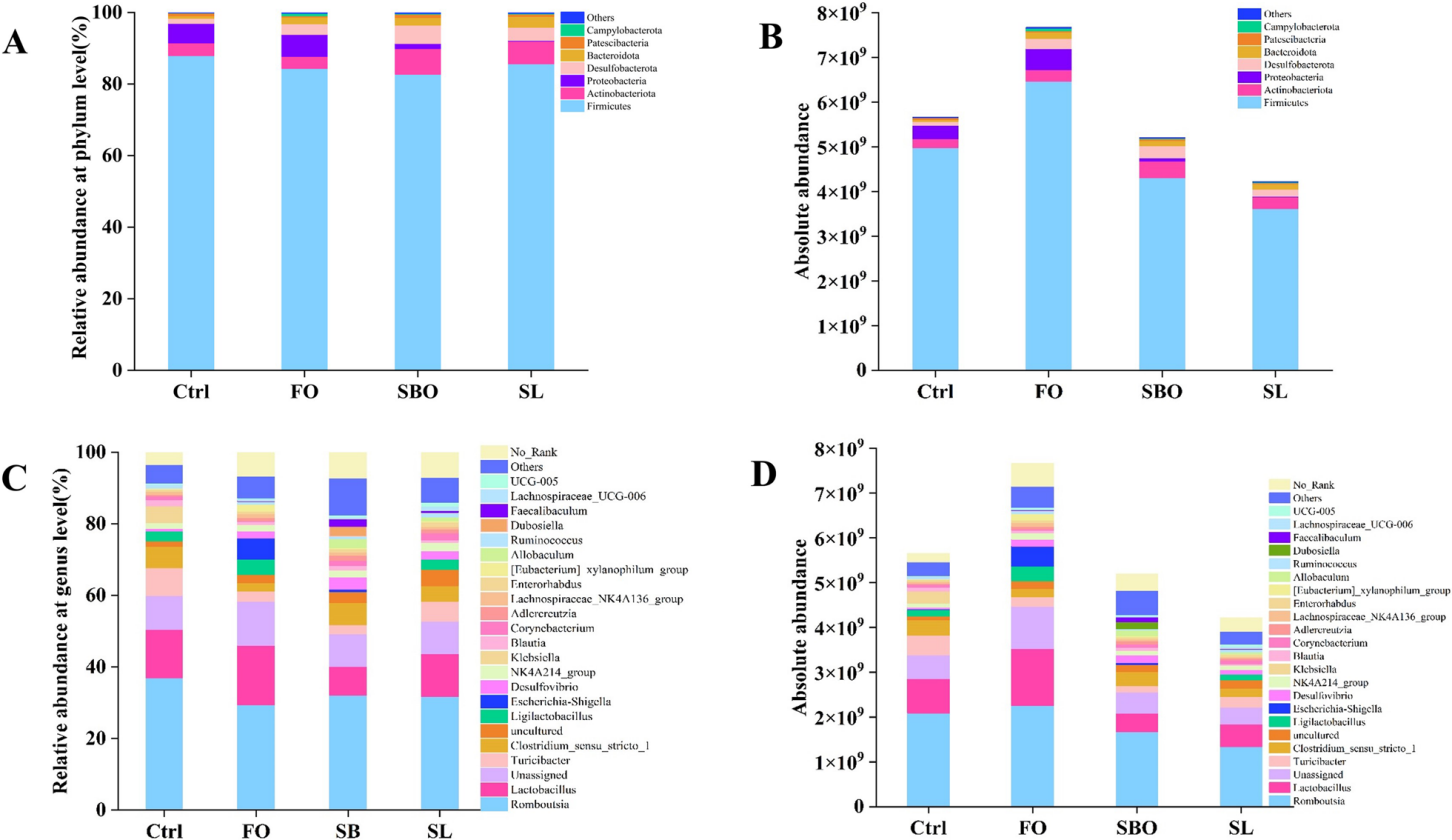
Histogram of structure at the level of Phylum and Gennus. **A** Relative abundance; **B** Absolute abundance; **C** Relative abundance; **D** Absolute abundance

The dominant bacteria identified at the phylum level in the collected door sample were *Firmicutes*, *Actinomyces*, *Proteobacteria*, *Desulphuricobacteria*, *Bacteroidetes*, *Patescibacteria* and *Campylobacter* (Fig. 6A). Among them, *Firmicutes*, *Actinomycetes* and *Proteobacteria* were found to be the most abundant. The average relative abundance of *Firmicutes* was highest in the Ctrl group (87.94%), followed by *Proteobacteria* (5.45%) and *Actinobacteria* (3.54%). In the FO group, the average relative abundance of *Firmicutes* (84.37%), *Proteobacteria* (6.12%) and *Actinobacteria* (3.36%) was highest. The top three bacteria in SBO group and SL group were *Firmicutes*, *Actinobacteria* and *Desulphurobacteria* respectively. Among them, *Bacteroidota* had the highest number in Ctrl group (87.94%), while it had the lowest number in SL group (3.03%). As shown in Fig. 6**,** there was a similar dominance of bacteria at the gate level among Ctrl, FO, SBO and SL groups. Interestingly, we found that *Firmicutesto* count was highest in FO group whereas significantly lower than Ctrl for SBO and SL groups (Fig. 6B). Compared to Ctrl group *Bacteroidetes* to count increased for FO, SBO and SL groups, the ratio offirmicutes to *Bacteroidetes* (F/B) was 126.97 for Ctrl group which was significantly higher than that for SL group (the lowest being 28.30). Abundance increased significantly for FO, SBO and SL groups compared to Ctrl.

##### Genus level

At the genus level (Fig. 6C), there were no significant differences in the proportion of major flora among different dietary groups compared to the control group. The proportion of major flora mainly consisted of *Romboutsia*, *Lactobacillus*, *Unassigned*, *Turicibacter*, *Clostridium_sensu_stricto_1* and *Ligilactobacillus*. Through absolute quantification (Fig. 6D), we found that the FO group showed an increased abundance of *Romboutsia*, Lactobacillus, *Ligilactobacillus*, *Escherichia-Shigella* and *Lachnospiraceae_NK4A136_group* compared to the Ctrl group. Specifically, *Desulfovibrio and NK4A214_group* significantly increased in abundance while *Klebsiella* and *Blautia* both decreased significantly in the FO group. Among them, *Blautia* exhibited the most pronounced decline in the SL group. Additionally, it is worth noting that with regard to *Blautia* in SL group declining the most.

### Categorical biomarker analysis

**Fig. 7:**
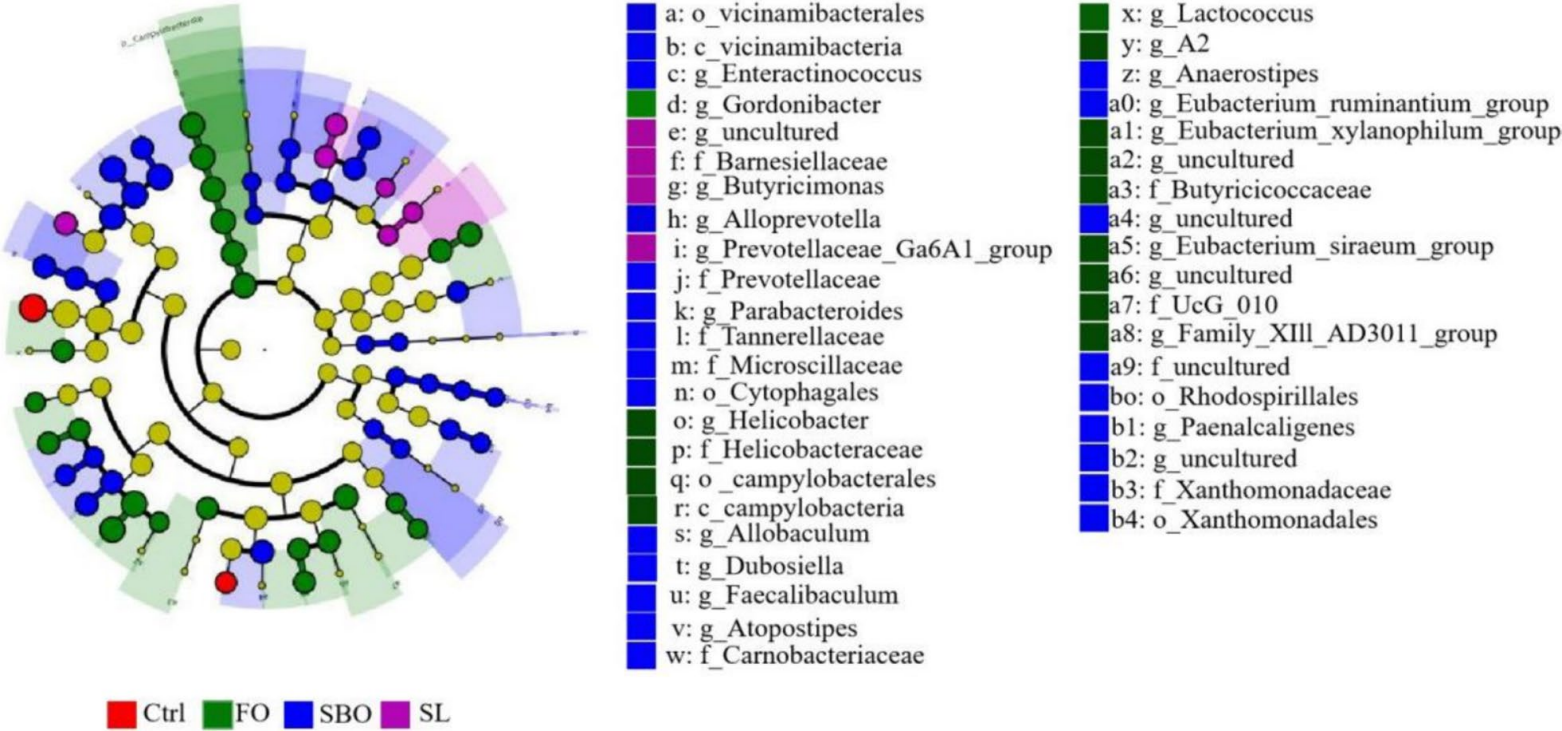
LEfSe branching diagram. Different colors repr; sent different groups. For instance, red nodes in branches indicate species with significantly higher abundance in the red group, green nodes indicate species with significantly higher abundance in the green group, and yellow nodes represent species with no significant difference in abundance between groups. The diameter of each node is proportional to its abundance. The nodes of each layer depict phyla, class, order, family, genus, species from the inside out, while the annotations on each layer indicate phyla, class, order, family, genus, species from the outside in. Annotations at the gate level are displayed on the outermost ring, whereas annotation information at other levels is provided in the legend on the right

**Fig. 8:**
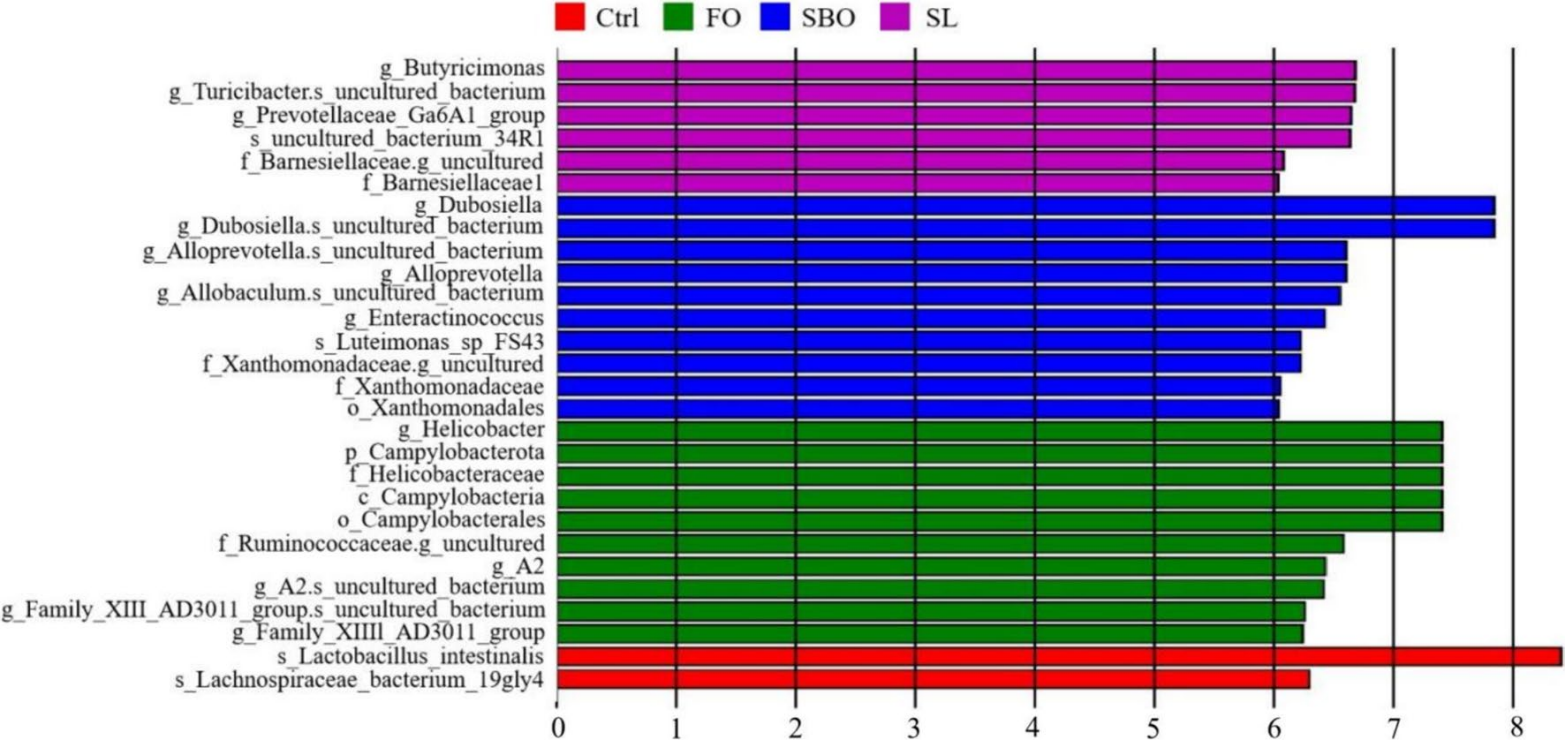
LDA score chart. The bars of various colors represent different species with an LDA score (log10) greater than 2 in distinct groups and significantly high abundance within each group. The length of the bar indicates the magnitude of the LDA score value

To further investigate the differences in intestinal microbiota among the Ctrl, FO, SBO, and SL groups, we employed the linear discriminant analysis effect size (LEFSe) (Fig. 7) and Linear Discriminant Analysis (LDA > 6) (Fig. 8) methods to identify specific taxa that were differentiated at various classification levels within each group. In the Ctrl group, two dominant bacterial groups were observed, *Lactobacillus_intestinalis* and *Lachnospiraceae_bacterium_19gly4*. The FO group exhibited ten dominant bacterial groups, including *Helicobacter*, *Campylobacterota*, and *Helicobacteraceae*, *Campylobacteria* with *Campylobacterales* showing the highest score. Similarly, the SBO group displayed ten dominant bacteria such as *Dubosiella* and *Dubosiela.s_uncultured_bacterium* which also scored high on LDA. Lastly, in the SL group, six dominant bacterial groups were identified including *Butyricimonas* and *Turicibacter.s_uncultured_bacterium* which also scored high on LDA. These findings indicated that specific bacterial taxa identified at different classification levels varied among the treatment groups.

## Discussion

In recent, numerous studies had reported the effects of different dietary lipids on the intestinal microbiota. However, the information of the effects of SBO, FO, and SL on serum and intestinal microbiota disorders was limited. This study demonstrated that SBO, FO, and SL possessed the potential to ameliorate dyslipidemia, regulated SCFAs concentration in the colon, and modulate intestinal microbiota in *SD* rats. Specifically, SBO, FO, and SL can inhibit serum LDL-C and HDL-C levels while altering intestinal microbiota by modifying the richness and diversity of microbial communities.

The variation in fatty acid composition among different dietary lipids can have diverse effects on the lipid profile. Previous studies had demonstrated that SBO and sea buckthorn seed oil possess the ability to reduce levels of TG, LDL-C, and TC in the serum of C57BL/6J mice, which may be attributed to the presence of palmitoleic acid in SBO [25]. Linoleic acid is believed to contribute to lowering blood cholesterol levels [26]. Previous study had shown that dietary lipids were rich in linoleic acid, such as corn oil, positively influence plasma TC and LDL-C levels. Oleic acid was found to have no significant impact on plasma TC levels, similar to other monounsaturated fatty acids, our results were in agreement with them. Furthermore, our findings revealed that rats fed with the SL diet had significantly lower serum LDL-C levels compared to Ctrl groups, possibly due to their higher content of polyunsaturated fatty acids [26].

The composition and stability of the intestinal microbiota were influenced by dietary and environmental factors, with dietary factors playing a crucial role [27]. Various diets can significantly impact the diversity and structure of the gut microbial community. Specifically, a high-fat Western diet disrupts the intestinal microbiota community, leading to chronic non-infectious and immune-related diseases [28]. From the result of the effects of three different dietary lipids on the intestinal microbiota of *SD* rats, we observed that compared with normal rats, the intestinal microbiota abundance of *SD* rats increased after FO diet, and the intestinal microbiota abundance of SBO and SL groups decreased, possibly because SBO and SL contain higher levels of saturated fatty acid. Notably, an increase in microbial diversity was also observed. PCoA and NMDS analysis clearly demonstrated significant deviations between clusters formed by Ctrl and SBO groups as compared to FO and SL groups. However, there was an overlap between FO and SL groups indicating greater similarity in bacterial colony composition. Additionally, it can be seen that there was a maximum distance between Ctrl and SBO groups suggesting significant differences in their respective intestinal microbiotas.

The composition of the intestinal microbiota in rats fed different dietary fats was significantly distinct from that of the normal group. At the phylum level, based on relative abundance analysis, consistent trends were observed with previous research in several major phyla within the SBO group [25]. Interestingly, absolute abundance analysis revealed significant variations in the absolute abundances of various phyla between groups (Additional file 1: Table S1). Absolute quantitative analysis evaluates changes in intestinal microbiota based on absolute abundance rather than relative abundance, reducing false-positive and false-negative rates during analyses [29]. Previous studies have demonstrated that maintaining a balanced F/B ratio was crucial for gut microenvironment homeostasis as it reflects gut microbial structure and health [30, 31]. Moreover, an increased F/B ratio is often considered indicative of obesity [32]. In this study, the Ctrl group exhibited the highest F/B ratio at 126.97. The F/B ratios of rats fed different dietary fats were significantly reduced, with the SL group displaying the lowest value at 28.30, suggesting positive effects on rat health following intervention with diverse dietary lipid diets. In contrast, *Desulfovibrio* and *NK4A214_group* both exhibit an increase in absolute abundance across various dietary treatment groups following intervention with different dietary lipid diets, while *Klebsiella* and *Blautia* both demonstrate a decrease in absolute abundance. Compared to the other three groups, the FO group displayed the highest absolute abundance of *Romboutsia*, *Lactobacillus*, *Escherichia-Shigella*, and *Lachnospiraceae_NK4A136_group*. The SL group shows the lowest absolute abundance of *Blautia* and *Lachnospiraceae_NK4A136_group*. These changes at the genus level suggest that dietary fat content selectively impacts the human gut microbiota, which may have clinical implications for healthy young adults.

SCFAs such as acetic acid, butyric acid, and propionic acid, are produced by the intestinal microbiota through the metabolism of complex carbohydrates and plant polysaccharides. SCFAs serves as a vital energy substrate for both humans and microorganisms [33]. Compared to the control group, different dietary interventions resulted in decreased levels of butyric acid and other short-chain fatty acids in colon contents due to reduced carbohydrate intake. High-sucrose and high-fat diets had been reported to decrease microbial diversity and abundance of health-beneficial bacteria that produced butyric acid. This study demonstrated that while there was no significant reduction in butyric acid concentrations among different groups compared to the Ctrl group, intake of SBO, FO, and SL significantly decreased acetic acid, propionic acid, and isovaleric acid, this could be attributed to variations in lipid types, dietary patterns, and doses which can influence SCFA concentrations in colon contents. Further investigations indicated that the F/B ratio may contribute to differences in SCFA concentrations within colon contents or fecal samples [31]. Therefore, it can be concluded that different dietary lipid interventions alter intestinal microbiota structure leading to changes in colon SCFA concentration. The results were consistent with previous research [34]. As mentioned above, this may be because oral force-feeding tubes containing 15% dietary lipids could negatively impact intestinal microbiota structure thereby affecting SCFA production [35–38]. The dominant short-chain fatty acids found in the colon contents across all groups were acetic acid, propionicacid, and butyricacid accounting for more than 90% of the total SCFAs [39, 40].

Our study showed that a fat-free diet could reduce blood fat and cholesterol levels to some extent. Compared with the Ctrl group, SBO could reduce blood lipid levels to a certain extent. Compared with other dietary fat groups, SL group could increase the abundance of *Bacteroides* in rats, fat-free diet could decrease the abundance of *Desulfovibrio* in rats, and FO could increase the abundance of *Romboutsia* in rats, indicating that the addition and type of dietary fat could affect the distribution of intestinal flora in rats to some extent. Therefore, we could try to choose some fats with a higher content of unsaturated fatty acids, similar to fish oil, while appropriate intake of SL to improve the abundance of intestinal microbiota.

## Conclusion

In summary, this study investigated the effects of three dietary lipids on the lipid profile, colon short-chain fatty acids, and intestinal flora of *SD* rats through a 6-week oral force-feeding experiment. The results revealed that the SL group had higher levels of serum triglycerides, while the FO group exhibited higher levels of serum total cholesterol. The dietary treatment groups exhibited significant reductions in serum levels of high-density lipoprotein cholesterol and low-density lipoprotein cholesterol. Detailed analysis indicated that the control group had higher contents of colon SCFAs compared to different dietary treatment groups. Furthermore, 16SrDNA sequencing results showed that all three dietary lipids could increase the diversity of intestinal microbiota. And the FO diet could enhance the abundance of intestinal microbiota. SL group had the most significant impact on *bacteroides*, the administration of SL group had a pronounced impact on the preservation of intestinal homeostasis and maintenance of normal function.

## Materials and methods

### Materials and chemical reagents

Sea buckthorn oil was extracted from sea buckthorn fruit by organic solvent extraction. Fish oil was provided by Wilmar International Ltd. (Shanghai, China). Structured lipid: The target compound was prepared in a reaction tank for 10 h at 50 °C with a molar ratio of 1:1. Novozym 435 (specific activity, 10,000 propyl laurate unit (PLU)/g). The Supelco 37 Component FAME Mixture was purchased from Sigma-Aldrich China (Shanghai, China). The gas chromatography grade acetic, propionic, isobutyric, butyric, isovaleric and valeric acids standards were all purchased from Macklin Regent Inc. (Purity ≥ 99.5%, Shanghai, China). All other chemicals were either of analytical or chromatographic grade, and were purchased from Sinopharm Chemical Reagent Co. Ltd. (Shanghai, China) and Thermo Fisher Scientific (Waltham, MA, US). Milli-Q water (Milli-Q Direct 8, Millipore, USA) was used for the solution preparation.

### Animal cultivation maintenance and designing

Animals were used with approval from South-Central MinZu University’s Ethics Committee in all cases (Animal protocol number 2022003). Twenty male *SD* rats (5 weeks old) were purchased from Shanghai SLRC Laboratory Animal Co., LTD., China, and raised in specific pathogen free Animal Center, Central South University for Nationalities (Certificate No: SYXK (Hubei) 2021-0089). After 7 days of adaptation, the rats were fed normal diet for another 2 weeks until adulthood (8 weeks of age, 302.23 ± 15.11 g), and were randomly divided into 4 groups (five rats in each group), namely control group (sterile normal saline), Ctrl group, SBO group, FO group and SL group. Rat in each group were given intragastric administration of normal saline, SBO, FO and SL once every 2 days for 42 consecutive days. The average diet intake of each rat was taken, and the dietary lipid oral gavage dosage was 15% of their average daily total normal diet intake. The samples of normal saline and lipid were respectively incubated in a warm bath at 37 °C for 20 min and then gavage. *SD* rats were subjected to light and dark cycling for 12 h under low stress conditions (24 ± 2 °C, 50 ~ 60% humidity, low noise), and were freely fed normal feed (Xiaoshu You Tai Biotechnology Co., LTD., Beijing) and sterile water. Weigh each cage of normal diet every two days, replaced with a new fresh normal diet, and add sterile water. Oral intragastric administration and water change were performed at fixed times. The rats were well cared for and their general health were monitored.

### Colonic content and colon tissue collection

In the last day of the 42-day experiment period, fasting for 12 h, intraperitoneal injection of 1% pentobarbital sodium solution (40 mg/kg, wt%) for anesthesia and cervical dislocation. The rats were dissected, colon contents and liver tissues were carefully collected and stored in sterile EP tubes. All samples were frozen in liquid nitrogen and kept at − 80 °C until the analysis was completed.

### Fatty acid analysis of lipid samples

Fatty acid analysis was performed using a gas chromatography system (Thermo, Trace 1300) with an automatic sampler (ISQ 7000, Thermo Scientific, China), a capillary gas chromatographic column (TG WAX 30 m × 0.25 mm i.d. × 0.25 μm), and a flame ionization detector. Fatty acid methyl esters were prepared according to previous research methods, and the GC analysis protocol was based on previous studies and slightly modified [34]. A simple summary is as follows: the initial oven temperature was maintained at 50 °C for 1 min, then increased to 200 °C at 25 °C /min, then increased to 230 °C at 5 °C /min and maintained for 15 min. The temperature of the injector and the FID detector was set to 250 °C and 280 °C respectively. The analysis lasted 28 min. The helium flow rate was 1.0 mL/min, the shunt ratio was 10, and the sample size was 1 μL [41]. All fatty acids were identified by comparison of retention time with FAME standard solution. All fatty acids were identified by comparing their retention times with those of FAME mixture standards. The relative contents of fatty acids were calculated by area normalization method.

### Serum sample preparation and analysis

Blood was rapidly collected through the orbital fossa during anesthesia, then centrifuged (1000 × g, 10 min, 4 °C) to collect serum, and isolated serum samples were stored at − 80 °C prior to analysis. Serum TG, TC, high density lipoprotein, and low density lipoprotein levels were rigorously analyzed using commercial test kits purchased from the Nanjing Institute of Jiancheng Bioengineering (Nanjing, China). All measurement protocols followed the instructions provided by the manufacturer.

### Determination of short-chain fatty acids in colonic contents

The SCFAs analyses referred to and modify previous studies [42]. Gas chromatography system (Nexis GC-2030, Shimadzu, Japan), Automatic sampler (AOC-20i Plus, Shimadzu, Japan), Flame Ionization Detector (FID) and High Resolution Capillary Gas Chromatography column (DB-FFAP 30 m × 0.25 mm i.d. × 0.25 μm) (Agilent, CA, USA). The initial temperature of the oven was 100 °C for 5 min. It then rised to 150 °C at 5 °C /min. It then heated up to 240 °C at 30 °C/min for 30 min. The temperature of the injector was 240 °C, and the temperature of the detector was 250 °C [34]. The carrier gas was high purity helium (He) with a constant flow rate of 1.0 mL/min. The shunt ratio was 75 and the sample size was 1 μL. The mixed standard was gas chromatography-grade acetic acid, propionic acid, butyric acid, isobutyric acid, valeric acid, isovaleric acid. The retention time of SCFAs in the sample of large intestine contents was compared with that of the standard curve to identify short-chain fatty acids, and the concentration of each short-chain fatty acid was calculated (μmol/g).

### intestinal microbiota analysis

Cecal contents were collected to extract microbiome genomic DNA. Genesky Biotechnologies Inc., Shanghai, 201315 (China) performed relative and absolute quantification of 16S rRNA gene pyrosequencing on the extracted DNA samples. For each sample, the V3-V4 region of the bacterial 16S ribosomal RNA gene was selected for amplification, an appropriate proportion of spike-in internal reference DNA was added to each sample, and a positive control sample was set and sequenced on Illumina MiSeq. The labeled sequences contain conserved regions identical to the natural 16S rRNA gene and artificially variable regions different from nucleotide sequences in the public database, they work similarly to internal standards and allow absolute quantification of the sample.

### Statistical analysis

Each experiment was performed at least three times and data were expressed as mean ± standard deviation. Statistical comparisons between groups were performed by one-way analysis of variance (ANOVA) with SPSS 19.0, followed by Duncan’s Post-Hoc test, with the significance level set at *p* < 0.05. The bar charts were draw using GraphPad Prism 7.0 and Origin 2019.

### Supplementary Information

Additional file 1.

## Data Availability

All data generated or analyzed during this study are available in this published article and its Additional files.
