# Supplementary material for: Revealing the effect of sea buckthorn oil, fish oil and structured lipid on intestinal microbiota, colonic short chain fatty acid composition and serum lipid profiles in vivo
Source: Nat Prod Bioprospect. 2024 Jul 3;14(1):41. doi: 10.1007/s13659-024-00461-z (PMC11219638; doi:10.1007/s13659-024-00461-z)
Supplement: Supplementary file 1 — Additional file 1. [file 13659_2024_461_MOESM1_ESM.docx]

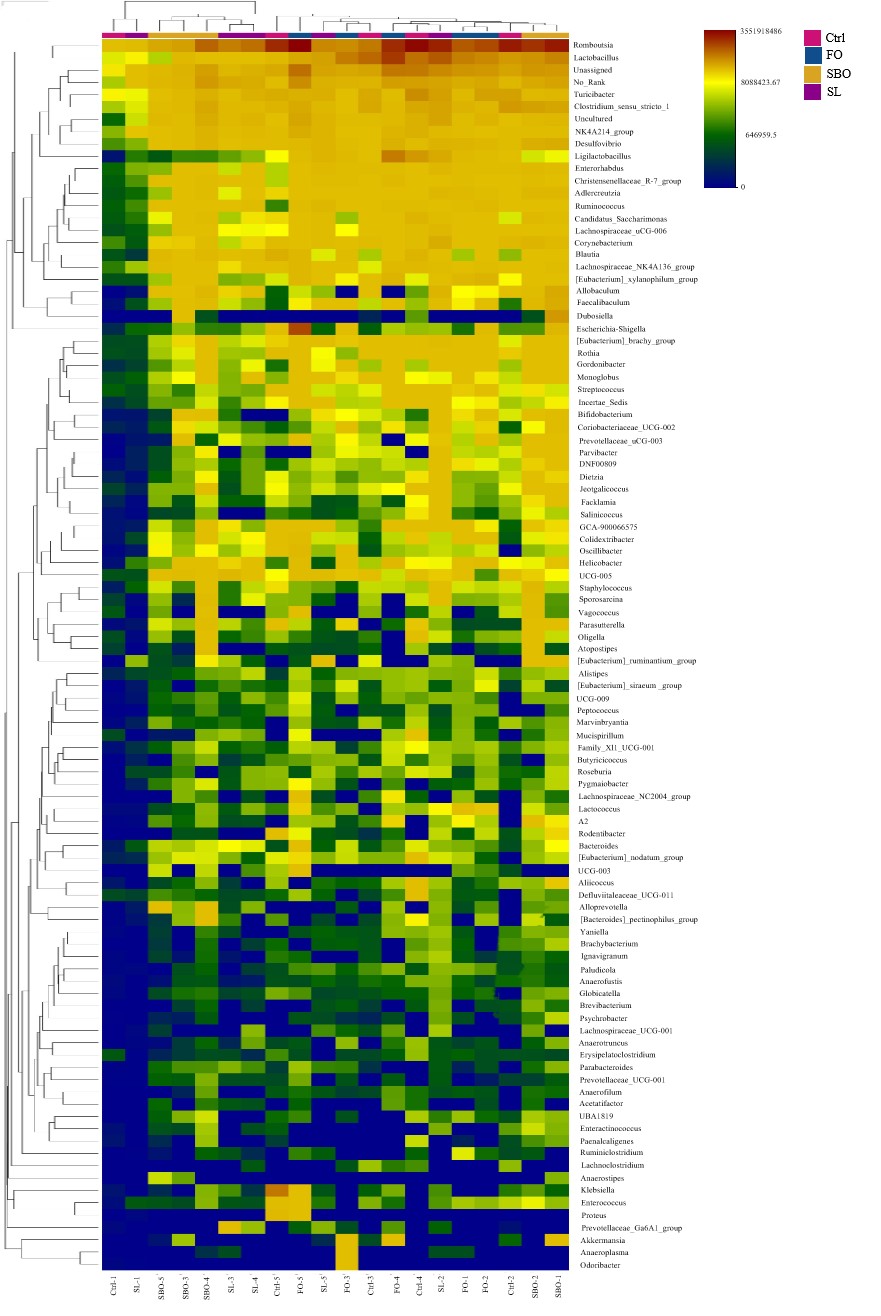


**Fig S1.** Each annotation level Heatmap. The horizontal coordinate represents the samples, while the vertical coordinate represents the 100 species with the highest abundance at this classification level. The colors in the figure indicate the abundance of each species, and the gradual change from blue to red signifies an increase in species abundance.

**Table S1**. Absolute abundance of seven major phyla at phyla level

|  | *Firmicutes* | *Actinobacteriota* | *Proteobacteria* | *Desulfobacterota* | *Bacteroidota* | *Patescibacteria* | *Campylobacterota* |
| --- | --- | --- | --- | --- | --- | --- | --- |
| Ctrl | 4.97×10^9^ | 2.00×10^9^ | 3.08×10^8^ | 8.07×10^7^ | 3.92×10^7^ | 4.34×10^7^ | 4.21×10^6^ |
| FO | 6.46×10^9^ | 2.58×10^8^ | 4.69×10^7^ | 2.29×10^8^ | 1.39×10^8^ | 3.14×10^7^ | 5.43×10^7^ |
| SBO | 4.30×10^9^ | 3.73×10^8^ | 4.60×10^7^ | 2.65×10^8^ | 1.11×10^8^ | 4.93×10^7^ | 1.04×10^7^ |
| SL | 3.61×10^9^ | 2.66×10^8^ | 8.76×10^5^ | 1.55×10^8^ | 1.28×10^8^ | 2.68×10^7^ | 9.30×10^6^ |
